# Feasibility and Efficacy of Percutaneous Lateral Lumbar Discectomy in the Treatment of Patients with Lumbar Disc Herniation: A Preliminary Experience

**DOI:** 10.1155/2015/378612

**Published:** 2015-01-28

**Authors:** Wenjin Jiang, Bolin Sun, Qirui Sheng, Xuepeng Song, Yanbo Zheng, Ligang Wang

**Affiliations:** Department of Interventional Radiology, Yantai Yuhuangding Hospital, Yantai, Shandong 264000, China

## Abstract

*Objective*. This study was aimed at evaluating the effectiveness and safety of percutaneous lateral lumbar discectomy (PLLD) in treating patients with lumber disc herniation. *Methods*. A total of 183 patients with lumbar disc herniation were recruited to receive PLLD surgery from April 2006 to October 2011. All the adverse effects were recorded during the follow-up at 1, 3, 6, and 12 months after PLLD. The clinical outcomes were determined by visual analog scale and Japanese Orthopaedic Association score. *Results*. The surgery was performed successfully in all patients (102 males and 81 females aged from 21 to 66 years) with a mean 16.6-month follow-up (range from 26 to 65 months). No postoperative complications, including intestinal and vascular complications, nerve injuries, and postoperative infections, were associated with PLLD. At one month after surgery, visual analog scale (3.12±1.44 versus 6.76±2.31, *P*<0.05) was significantly lower than the baseline and was sustained until 24 months after surgery (3.25 ± 1.78 versus 6.76±2.31, *P*<0.05). Besides that, Japanese Orthopaedic Association score (25.25±3.21 versus 11.78±2.38, *P*<0.05) was increased when compared to the baseline. *Conclusions*. PLLD was a promising, mini-invasive, and effective treatment for lumber disc herniation.

## 1. Introduction

Lumbar disc herniation (LDH), characterized by pain and numbness in lower extremity (e.g., radicular unilateral or bilateral leg pain) [1], is one of the most common causes of nerve root pain and severely affects life quality of working adults [2]. It is estimated that 1-2% of people would be troubled by LDH [3, 4]. The underlying mechanism of LDH is disc degeneration or trauma which induces the translocation of nucleus (annulus fibrosis) into the vertebral canal, forming the nucleus pulposus and compressing the spinal cord nerves [5, 6]. Therefore, removal of nucleus pulposus would alleviate the LDH symptoms.

Traditionally, open surgery is the standard method for herniation removal in LDH patients with persistent low-back pain [7]. However, due to the limitations of open surgery such as high perioperative complication and expensive fee, percutaneous lumbar discectomy (PLD) has emerged and been widely applied as an optional choice for LDH treatment [8, 9]. PLD can achieve disc decompression through physically removing partial disc nucleus pulposus materials with 70 to 90% success rate [10, 11]. However, PLD is unable to remove the whole herniated nucleus pulposus tissues due to the angle of puncture. Therefore, classical PLD is localized to some contained LDH while there was low efficacy when applied in the cases with damage to annulus fibrosus, protrusion, or extrusion [12]. To overcome these limitations, we amended the angle of puncture of classical PLD and named it percutaneous lateral lumbar discectomy (PLLD). A total of 183 patients had been successfully treated with PLLD from April 2006 to October 2011. In the present study, we aimed to evaluate the clinical efficacy and safety of PLLD.

## 2. Patients and Methods

### 2.1. Patient Profile

Between April 2006 and October 2011, a total of 183 patients with LDH were screened to undergo PLLD treatment. The eligible criteria were as follows: (1) having sciatic and/or low-back pain; (2) duration of symptoms ≥2 months; (3) having LDH with damage to annulus fibrosus, protrusion, or extrusion; (4) herniated tissues with a protrusion less than half of anteroposterior diameter of spinal canal; (5) no osseous spinal canal stenosis; (6) symptoms and physical findings which should be confirmed by computed tomography (CT) or magnetic resonance (MR) imaging; (7) agreement to receive PLLD and complete the follow-up.

The exclusion criteria included (1) age < 18 years; (2) having serious life-threatening diseases; (3) not being able to tolerate a procedure under local anesthesia; (4) previously having a history of open lumbar surgery; (5) previously having rectum or bladder problems; (6) previously having a progressive neurologic deficit or cauda equina syndrome, pregnancy, infection, or intraspinal tumor; (7) previously having any other cause of pain as revealed by CT or MR imaging.

The study protocol was approved by the Ethics Committee of Yantai Yuhuangding Hospital. Written informed consent was obtained from each patient.

### 2.2. Surgical Instruments

**Figure 1:**
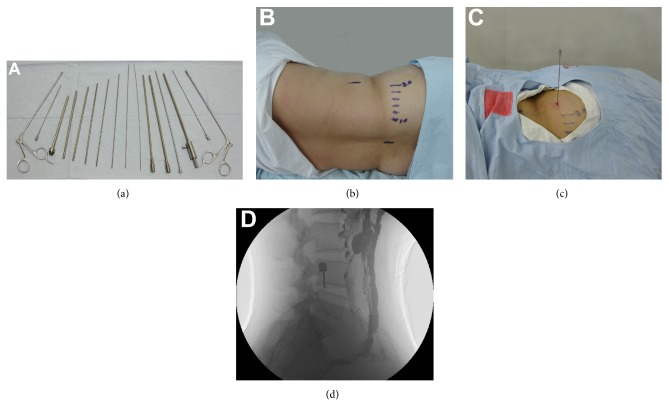
Surgical technique and procedures of PLLD surgery: (a) surgical instruments used for PLLD surgery; (b) the markers for indicating the skin entry site; (c) needle insertion was performed at the selected skin entry site; (d) the view of the relative position between the intestines and the intervertebral space under fluoroscopy.

The surgical instruments, provided by Hangzhou Tonglu Medical Instrument Company (Hangzhou, China), included an 18-gauge hollow puncture needle, a guide wire, five expanding cannulas, a working cannula, a suction irrigation device, and two nucleus pulposus forceps (Figure 1(a)).

### 2.3. Surgical Procedures

Before the surgery, the patient needed to be carefully preparing the bowel and emptying the bladder. Briefly, the patients received a dilute barium meal under X-ray, which was introduced through the rectum to fill the left or entire colon with a retention time of 10 min. The patient was asked to keep the contralateral (unaffected side) lateral position (Figure 1(b)). Fluoroscopy in the anterior-posterior (AP) and lateral views was used to precisely locate the targeted disc.

The point of the puncture was located in the area between the posterior axillary line and midaxillary line (Figure 1(c)). After local anesthesia, the hollow puncture needle was advanced behind the colon to the rear one-fourth of the intervertebral space of herniated disk. The needle position was visualized under fluoroscopy and the angle of needle was adjusted in order to avoid any colon damage (Figure 1(d)). During the needle insertion, patients were instructed to perform diaphragmatic breathing. The skin entry point was enlarged using a blade after inserting the guide wire into the disc space. The dilator was introduced over the guide wire into the intervertebral space and then a working cannula was inserted into the margin of the disc space over the dilator. After that, a trephine was inducted into the nucleus pulposus cavity to cut annulus fibrosus. Then, nucleus pulposus forceps were used to grasp and remove the herniated disc material followed by irrigation and drainage with cold saline solution of disc cavity. The working cannula was then withdrawn and the incision was sutured.

After surgery, patients remained in bed without position restriction for three days. On the fourth day, patients were encouraged to perform moderate exercise of the lumbar muscles. Fourteen days after operation, exercise for lower extremities was encouraged.

### 2.4. Clinical Outcomes

Pain intensity and neurological improvement were the primary outcome variables of this study. The pre- and postoperative visual analogue scales (VAS) were recorded to assess the pain intensity [13, 14]. Neurological improvement was assessed by the Japanese Orthopaedic Association (JOA) score [15, 16]. The JOA score recovery rate was calculated using the following formula: recovery rate = [(postoperative score − preoperative score)/(29 − preoperative score)] × 100%. The recovery rate was ranked as Excellent (>90%), Good (75–89%), Fair (50–74%), or Poor (<50%). The effective rate was defined as the percentage of the cases with Excellent and Good recovery.

### 2.5. Follow-Up

Postoperative follow-up was performed by an experienced nurse at 1, 3, 6, and 12 months and thereafter annually. Patients were interviewed either by telephone or in clinics. Computed tomography (CT) and/or magnetic resonance imaging (MRI) of lumbar vertebra were carried out at least once. VAS and JOA scores were recorded for each follow-up assessment.

### 2.6. Statistical Analysis

Statistical analysis was performed with SPSS 15.0 software (SPSS Inc., Chicago, IL). The values of VAS and JOA score were tested for normality distribution using Kolmogorov-Smirnov test and presented as mean ± standard deviation (SD). Student's* t*-test was applied to evaluate the improvement in VAS and JOA score. *P* < 0.05 was considered statistically significant (two-tailed).

## 3. Results

### 3.1. Clinical Characteristics of Patients

The basic characteristics of the patients enrolled in this study were summarized in Table 1. The duration of symptoms varied from 6 months to 21 years. Major clinical symptoms included pain in the waist and hip, radicular pain, numbness, paresthesias, muscle atrophy in the lower extremities, and claudication. Furthermore, foot drop was observed in ten cases and pelveo-perineal dysesthesia and/or sphincter disturbances were observed in two cases. All patients had damage to annulus fibrosus or disc protrusion and extrusion; conservative treatment for more than 6 months was ineffective before the surgery.

### 3.2. Perioperative and Early Outcome of Patients Undergoing PLLD Treatment

**Figure 2:**
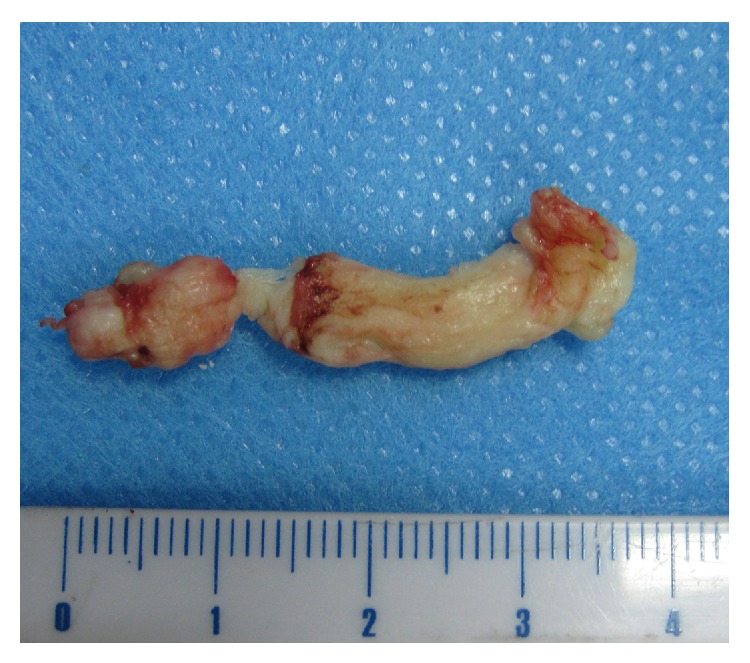
A representative nucleus pulposus removed by PLLD surgery with the length longer than 4 cm.

**Figure 3:**
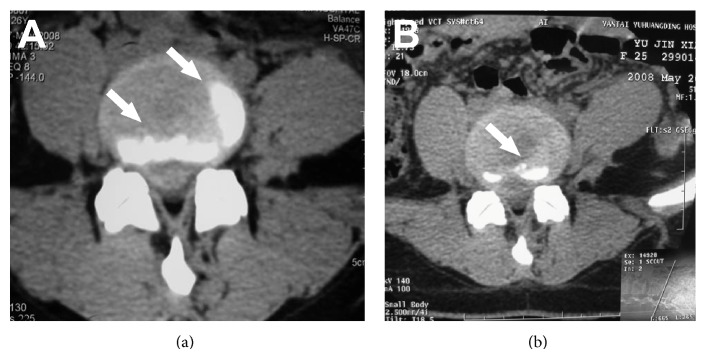
(a) Pre- and (b) postoperative CT images of a representative patient with L4-5 intervertebral disc herniation (the protrusion is indicated by white arrows); protrusion was reduced significantly 1 week after the operation (as indicated by white arrows).

The PLLD surgery was successfully performed in all 183 patients with a mean of 56 minutes of operation time (ranging from 40 to 120 minutes). The parameters of surgery and incidence of perioperative complications were shown in Table 2. In 59 out of 183 cases (32.2%), the herniation tissues removed by PLLD surgery had a length over 1 cm (Figure 2) with a mean weight of 3.7 g (ranging from 3 to 5 g). At 1 week after surgery, protrusion was significantly reduced (Figures 3(a) and 3(b)). At 1 month after surgery, the mean VAS was 3.12 ± 1.44, which was significantly lower than baseline (6.76 ± 2.31, *P* < 0.05). The mean JOA score was significantly higher than the baseline score (25.25 ± 3.21 versus 11.78 ± 2.38, *P* < 0.05). No intestinal, vascular, or nervous injuries were observed.

### 3.3. Short-Term Outcome of Patients Undergoing PLLD Treatment

The mean VAS and JOA score during follow-up were listed in Table 3. At the 12 and 24 months after surgery, the mean VAS was 3.25 ± 1.78 and 2.57 ± 1.64, respectively. Furthermore, the rate of Excellent, Good, Fair, and Poor scores of JOA was 58.5%, 34.4%, 3.8%, and 3.3%, respectively. Hence, the effective rate of PLLD was 92.9%. A total of 114 (62.3%) underwent imaging examination during the follow-up, of which 21 cases (18.4%) displayed the complete disappearance of the herniation, 87 (76.3%) had the significant reduction, and only 6 (5.2%) had no reduction of the herniation.

As shown in Table 4, 4 patients experienced postoperative complications without death. These complications were generally mild, and all fully resolved without sequelae. By the end of 24-month follow-up, only six patients underwent additional surgery.

## 4. Discussion

In the present study, our data indicated that PLLD was a safe and effective strategy to remove the herniated disc, which resulted in relieving pain and improving neurological function in patients with LDH. Compared with the classical PLD, there were two merits of PLLD. For the classical PLD, the skin entry point is about 8 to 12 cm lateral to the spinous process. Thus, the needle and working cannula are oriented approximately 30–45 degrees to the coronal plane [17]. It was difficult for the full removal of disc herniation. In PLLD surgery, the skin entry site was located between the posterior axillary line and midaxillary line and the insertion of needle was almost parallel to coronal plane. Besides that, the needle was advanced into the rear one-fourth of the intervertebral space of herniated disk in the PLLD. This location and depth of needle insertion facilitated the complete removal of disc herniation, thus relieving or even eliminating the related nerve compression syndromes. In this study, 183 patients had severe lumbar disc herniation with damage to annulus fibrosus or protrusion and extrusion types, which are usually considered contraindications to PLD [17, 18]. After treatment, an effective rate of 92.9% was achieved which was comparable with open surgery [19, 20]. Therefore, our data indicated that PLLD, as a minimally invasive treatment, may be effective in treating some severe lumbar disc herniation, lumbar disc prolapse, and spinal stenosis caused by disc bulge. However, the minimally invasive treatment failed to improve the outcome of patients compared with conventional microdiscectomy in one clinical trial [21]. The possible reason was that the minimally invasive approach was different from ours. Hence, it was necessary to conduct one head-to-head trial to compare the efficacy of PLLD with open surgery.

### 4.1. Anatomical Basis of PLLD Surgery

There are some important muscles, arteries, and organs (such as the psoas major muscle, lumbar arteries, kidney, and ureter) in the lateral region of lumbar disc. For lumbar arteries, their position is relatively constant to lumbar vertebra. For example, the L3 and L4 arteries arise from the dorsal side of the abdominal aorta while the L5 artery arises from the middle sacral artery, iliolumbar artery, or L4 artery. Lumbar arteries run backward and laterally around the sides of the bodies of the lumbar vertebrae. Between the transverse processes of the vertebrae, each lumbar artery divides into a dorsal and an abdominal branch. The dorsal branch also gives off a spinal branch to the interior of the spinal canal. However, the stems and branches of the lumbar arteries are located away from the intervertebral disc, which reduced the risk of vascular injury in PLLD surgery [22]. Another important organ is the kidney, the location of which should be investigated by radiologic imaging (CT and/or MRI) before surgery. The left kidney is approximately at the vertebral level T12 to L3 while the right is slightly lower. Therefore, PLLD can neither be used for the treatment of L1-2 and L2-3 disc herniation due to the adjacent position of kidney to L1-L2 and L2-L3 intervertebral discs nor be used for the treatment of L3-L4 disc herniation in the patients with a lower kidney position. Furthermore, the positions of ureter and intestine must be considered when performing PLLD surgery. Ureter, with a mean diameter of 0.5 cm, runs retroperitoneally and descends along the anterior aspect of psoas major muscle. Its anteroposterior projection usually overlaps with that of the distal end of the lumbar vertebral transverse process. Sigmoid colon is at the left region of the vertebral level L2 to L5, and ileum, cecum, and ascending colon are at the right region. However, ureter and the abovementioned segments of intestine generally have no overlap with the posterior one-third of the intervertebral disc. Therefore, the L3-L4 and L4-L5 intervertebral discs were considered the ideal targets for PLLD surgery. For avoiding the possible injury to nerves during the PLLD surgery, it was very useful to apply real-time electromyographic monitoring. According to the recent systematic review, the addition of electromyographic monitoring to the minimally invasive lateral transpsoas approach to the lumbar spine has contributed to decrease of the complication rate from 30% to less than 1% [23].

### 4.2. Precautions for PLLD Surgery

The precautions for PLLD surgery included the following: (1) bowel preparation (barium meal for filling the left or entire colon) should be performed in order to make the colon more visible under fluoroscopic monitoring; (2) the position of patients during the surgery should be the contralateral lateral position, which could avoid the possible needle insertion injury of intestine in case there was a position shift of the intestines to ventral midline due to gravity; (3) during the needle insertion, patients should be instructed to perform diaphragmatic breathing to reduce the risk of intraoperative intestinal injury.

Several limitations should be addressed regarding this study. This was an observational study in a single site and there was no control group. Hence, multiple center, randomized, and controlled trials are encouraged to test its safety and efficiency. The follow-up period was relatively short. This may contribute additional bias to the current study. Therefore, long follow-up is undergone to confirm the long-term outcomes.

In conclusion, PLLD was a promising strategy with great potential for patients with central or paracentral L3–L5 disc herniation due to its good safety and minimal invasion.

## Figures and Tables

**Table 1:** Baseline characteristics of the enrolled patients.

Characteristics	Value or number of patients (%)
Age, years	46 (21–66)
BMI (kg/m^2^)	23.1 ± 3.4
Male	102 (55.7)
Previous treatments of disk herniation	
PLD	15 (8.2)
Intervertebral discolysis with collagenase	4 (2.2)
PLDD	3 (1.6)
Open surgery^*^	4 (2.2)
Location of disk herniation	
Only L3-4	26 (14.2)
Only L4-5	143 (78.1)
L3-4 and L4-5	14 (7.7)

BMI: body mass index; PLD: percutaneous lumbar discectomy; PLDD: percutaneous laser disc decompression surgery.

^*^The surgery was performed in the different positions with LDH.

**Table 2:** Intraoperative characteristics.

Characteristics	Value or number of patients (%)
Operation time, min	56 (40–120)
Blood loss, mL	63.1 ± 53.4
Length of hospital stay, day	2.1 ± 1.3
Intraoperative complications	
Dural tear or spinal fluid leak	1 (0.5)
Nerve root injury	0
Other	2 (1.1)

**Table 3:** Primary outcome measurements in overall patients.

	Baseline	1 month	3 months	6 months	12 months	24 months
VAS score	6.76 ± 2.31	3.12 ± 1.44	3.36 ± 1.34	3.26 ± 1.23	3.25 ± 1.78	2.57 ± 1.64
JOA score	11.78 ± 2.38	25.25 ± 3.21	26.51 ± 2.15	27.98 ± 1.23	27.31 ± 1.87	27.45 ± 1.67

**Table 4:** Postoperative complications or events in overall patients within 2-year follow-up.

Postoperative complications	Number of patients (%)
Nerve root injury	1 (0.5)
Wound hematoma	1 (0.5)
Wound infection	2 (1.1)
Other	1 (0.5)
Additional surgery rate	
One year	2 (1.1)
Two years	6 (3.3)

## References

[1] Andersson G. B., Brown M. D., Dvorak J. (1996). Consensus summary of the diagnosis and treatment of lumbar disc herniation. *Spine*.

[2] Keller R. B., Atlas S. J., Singer D. E. (1996). The maine lumbar spine study, part I: background and concepts. *Spine*.

[3] Deyo R. A., Tsui-Wu Y. J. (1987). Descriptive epidemiology of low-back pain and its related medical care in the United States. *Spine*.

[4] Rhee J. M., Schaufele M., Abdu W. A. (2006). Radiculopathy and the herniated lumbar disc. Controversies regarding pathophysiology and management. *The Journal of Bone and Joint Surgery. American Volume*.

[5] Rihn J. A., Hilibrand A. S., Radcliff K. (2011). Duration of symptoms resulting from lumbar disc herniation: effect on treatment outcomes—analysis of the Spine Patient Outcomes Research Trial (SPORT). *Journal of Bone and Joint Surgery—Series A*.

[6] Chen M., Chen R., Xiong J., Yi F., Chi Z., Zhang B. (2011). Effectiveness of heat-sensitive moxibustion in the treatment of lumbar disc herniation: study protocol for a randomized controlled trial. *Trials*.

[7] Koes B. W., Van Tulder M. W., Peul W. C. (2007). Diagnosis and treatment of sciatica. *British Medical Journal*.

[8] Singh V., Benyamin R. M., Datta S., Falco F. J., Helm S., Manchikanti L. (2009). Systematic review of percutaneous lumbar mechanical disc decompression utilizing Dekompressor. *Pain Physician*.

[9] Singh V., Manchikanti L., Benyamin R. M., Helm S., Hirsch J. A. (2009). Percutaneous lumbar laser disc decompression: a systematic review of current evidence. *Pain Physician*.

[10] Kahanovitz N., Viola K., Muculloch J. (1989). Limited surgical discectomy and microdiscectomy. A clinical comparison. *Spine*.

[11] Onik G. M. (2000). Percutaneous diskectomy in the treatment of herniated lumbar disks. *Neuroimaging Clinics of North America*.

[12] Lee S.-H., Byung U. K., Ahn Y. (2006). Operative failure of percutaneous endoscopic lumbar discectomy: a radiologic analysis of 55 cases. *Spine*.

[13] Grant S., Aitchison T., Henderson E. (1999). A comparison of the reproducibility and the sensitivity to change of visual analogue scales, Borg scales, and likert scales in normal subjects during submaximal exercise. *Chest*.

[14] Reips U.-D., Funke F. (2008). Interval-level measurement with visual analogue scales in internet-based research: VAS generator. *Behavior Research Methods*.

[15] Clinical Outcomes Committee of the Japanese Orthopaedic Association SoEoBP, Subcommittee on Low Back Pain and Cervical Myelopathy Evaluation of the Clinical Outcome Committe of the Japanese Orthopaedic Association, Fukui M. (2007). JOA back pain evaluation questionnaire: initial report. *Journal of Orthopaedic Science*.

[16] Fukui M., Chiba K., Kawakami M. (2007). Japanese orthopaedic association back pain evaluation questionnaire. Part 2. Verification of its reliability: the subcommittee on low back pain and cervical myelopathy evaluation of the clinical outcome committee of the Japanese orthopaedic association. *Journal of Orthopaedic Science*.

[17] Teng G.-J., Jeffery R. F., Guo J.-H. (1997). Automated percutaneous lumbar discectomy: a prospective multi-institutional study. *Journal of Vascular and Interventional Radiology*.

[18] Hirsch J. A., Singh V., Falco F. J. E., Benyamin R. M., Manchikanti L. (2009). Automated percutaneous lumbar discectomy for the contained herniated lumbar disc: a systematic assessment of evidence. *Pain Physician*.

[19] Azimi P., Mohammadi H. R., Montazeri A. (2012). An outcome measure of functionality and pain in patients with lumbar disc herniation: a validation study of the Japanese Orthopedic Association (JOA) score. *Journal of Orthopaedic Science*.

[20] Matsumoto M., Watanabe K., Hosogane N. (2013). Recurrence of lumbar disc herniation after microendoscopic discectomy. *Journal of Neurological Surgery, Part A: Central European Neurosurgery*.

[21] Arts M. P., Brand R., van den Akker M. E., Koes B. W., Bartels R. H. M. A., Peul W. C. (2009). Tubular diskectomy vs conventional microdiskectomy for sciatica: a randomized controlled trial. *The Journal of the American Medical Association*.

[22] Shimizu S., Tanaka R., Kan S., Suzuki S., Kurata A., Fujii K. (2005). Origins of the segmental arteries in the aorta: an anatomic study for selective catheterization with spinal arteriography. *American Journal of Neuroradiology*.

[23] Uribe J. S., Vale F. L., Dakwar E. (2010). Electromyographic monitoring and its anatomical implications in minimally invasive spine surgery. *Spine*.

